# Knockdown of SERPINB2 enhances the osteogenic differentiation of human bone marrow mesenchymal stem cells via activation of the Wnt/β-catenin signalling pathway

**DOI:** 10.1186/s13287-021-02581-6

**Published:** 2021-10-07

**Authors:** Kai Hang, Li Ying, Jinwu Bai, Yibo Wang, Zhihui Kuang, Deting Xue, Zhijun Pan

**Affiliations:** 1 Department of Orthopedic Surgery of the Second Affiliated Hospital, School of Medicine, Zhejiang University, No. 88, Jiefang Road, Hangzhou, 310009 China; 2 Orthopedics Research Institute of Zhejiang University, No. 88, Jiefang Road, Hangzhou, 310009 China

**Keywords:** SERPINB2, Wnt/β-catenin, hBMSCs, Fracture healing

## Abstract

**Background:**

Globally, bone fractures are the most common musculoskeletal trauma, and approximately 8–10% of cases that fall into the categories of delayed or non-union healing. To date, there are no efficient pharmacological agents to accelerate the healing of bone fractures. Thus, it is necessary to find new strategies that accelerate bone healing and reduce the incidence of non-union or delayed fracture healing. Previous studies have revealed that the plasminogen activation system has been demonstrated to play an important role in bone metabolism. However, the function of SERPINB2 in the osteogenesis of hBMSCs remains unclear. Therefore, in this study, we investigated the effects and mechanism of SERPINB2 on osteogenic differentiation.

**Methods:**

We investigated the osteogenesis effects of hBMSCs by both exogenous SerpinB2 protein and SERPINB2 gene silencing in vitro. Cell proliferation assay was used to assess the effect of exogenous SerpinB2 or SERPINB2 silencing on proliferation of hBMSCs. qPCR and Western blotting analysis detected the expression of target genes and proteins respectively. ALP staining was used to evaluated ALP activity and Alizarin Red staining (ARS) was used to evaluate mineral deposition. In vivo, a murie tibial fracture model was established, histological evaluation and radiographic analysis was used to confirm the therapeutic effects of SERPINB2 silencing in fracture healing. Statistical significance between two groups was determined by Student’s t test, one-way ANOVA or Bonferroni’s post-hoc test according to the distribution of the tested population.

**Results:**

The addition of exogenous SerpinB2 protein inhibted osteoblast differentiation of hBMSCs in vitro, while SERPINB2 gene silencing significant promote osteoblast differentiation of hBMSCs in vitro. And silenced SERPINB2 gene also increased mineral deposits. Moreover, β-catenin levels were up-regulated by SERPINB2 gene depletion. And the enhancement of osteogenic differentiation induced by SERPINB2 silencing was almost inhibited by specific Wnt/β-catenin signaling pathway inhibitor. In a murine tibial fracture model, local injection of SERPINB2 siRNA improved bone fracture healing.

**Conclusions:**

Taken together, these findings indicate that SERPINB2 silencing promoted osteogenic differentiation of BMSCs via the Wnt/β-catenin signaling pathway, and silenced SERPINB2 in vivo effectively promotes fracture healing, suggesting that SERPINB2 may be a novel target for bone fracture healing.

**Supplementary Information:**

The online version contains supplementary material available at 10.1186/s13287-021-02581-6.

## Introduction

Globally, bone fractures are the most common musculoskeletal trauma according to the National Hospital Ambulatory Medical Care Survey performed in 2017 [1]. In the People’s Republic of China, the incidence rate of trauma fractures of the trunk, arms and legs is 3.2 per 1000 population in 2014. Healing fractures is a complex biological process impaired by several adverse factors, including sleeping less, aging, smoking, alcohol consumption, and history of diabetes [2–4]. The prevalence of hip fracture in China has an apparent geographic variation. In those 55 years and older population, the total number of hip fractures increased about fourfold with a rapid increase in the total costs for hospitalization [5, 6]. Advances in the study of biology have given us a better understanding of the fracture healing process at the molecular level; however, there are still approximately 8–10% of cases that fall into the categories of delayed or non-union healing. Treatment of impaired healing in these patients not only represents a tremendous burden on healthcare systems but also increases patients’ pain and financial burdens [7–9]. To date, there are no efficient pharmacological agents to accelerate the healing of bone fractures. Thus, it is necessary to find new strategies that accelerate bone healing and reduce the incidence of non-union or delayed fracture healing.

The postnatal fracture healing process shows a similar ontological process to skeletal development in embryology [10, 11]. There are two major bone repair pathways: (1) intra-membranous ossification (also known as primary healing), which occurs in bone fractures with minimal fracture gaps, in which osteogenic progenitor cells directly differentiate into osteoblasts; (2) endochondral ossification (also known as secondary healing) [12, 13], the most common healing type in diaphysis fractures which with unstable mechanics and large fracture gaps [14]. The endochondral ossification healing process is divided into three phases: inflammation response, callus formation, and callus remodeling [15]. In the inflammatory phase, a hematoma is formed after the fracture, and the immune system initiates and promotes bone repair and angiogenesis by recruiting cellular repair sources, including osteogenic precursor stem cells and angiogenesis factors, to the fracture site [16–18].

The concept of mesenchymal stem cells (MSCs) was first described by Caplan et al. in 1991, who combined the terms “stem” and “stromal” [19], Numerous studies showed that MSCs are distributed widely in adult or neonatal tissues, such as bone marrow (BMSCs), adipose tissue, and umbilical cord [19, 20]. MSCs are characterized by their self-renewal ability and multilineage differentiation. In terms of their multipotency, the use of MSCs is the ideal therapy intervention for severe diseases requiring regenerative repair. To date, hundreds of clinical trials have been conducted regarding MSCs [21–23]. Among MSCs, BMSCs are the most widely studied and are better documented in clinical trials [24]. Such trials in skeletal systems include studies on non-union fracture healing and osteogenesis imperfect [25].

Serpin Family B Member 2 (SerpinB2) is a member of the clade B subgroup of serine protease inhibitors (serpins), a superfamily of proteins [26, 27]. In contrast to the conventional serine protease inhibitor, SerpinB2 was first identified as a placental tissue-derived urokinase plasminogen activator (uPA) inhibitor, also called plasminogen activator inhibitor type 2 (PAI-2) [28]. SerpinB2 is expressed widely in various cells and tissues, including the bone marrow, bladder, skin, and lung [29–31]. Lacking a classic secretory signal, SerpinB2 was thought to be secreted in the cytoplasm [32], however, recent studies have shown that SerpinB2 reaches the extracellular space and exerts its inhibiting function as a uPA and tissue plasminogen activator (tPA) [31, 33]. Previous studies have demonstrated that inhibited plasminogen activation system promoted bone formation [34], uPA and tPA double knockout increased bone formation in mice. SERPINB2 is a TGFβ responsive gene and knockdown of SERPINB2 gene partially restored the osteoblastic differentiation potential of non-bone forming human bone marrow stromal stem cells (hBMSC^−Bone^) [35], which suggested that SERPINB2 gene may play an important role in the osteogenic differentiation of BMSCs.

In this study, we investigated the effects of exogenous SerpinB2 protein or SERPINB2 gene silencing on osteogenesis differentiation of hBMSCs. We found that SERPINB2 gene silencing promoted osteogenic differentiation of hBMSCs via the Wnt/β-catenin signaling pathway, and silenced SERPINB2 gene in vivo effectively promotes fracture healing, suggesting that SERPINB2 may be a novel target for bone fracture healing.

## Results

### Endogenous SerpinB2 expression and the influence of exogenous recombinant SerpinB2 on osteogenesis differentiation of hBMSCs

**Fig. 1:**
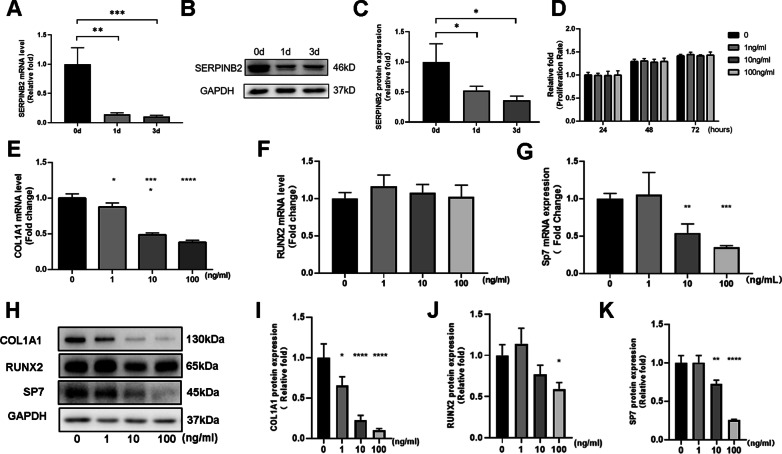
Endogenous SerpinB2 expression and the influence of exogenous recombinant SerpinB2 on the proliferation and osteogenesis differentiation of hBMSCs. **a**–**c** SerpinB2 expression level decreased during osteogenic differentiation of hBMSCs. **d** SerpinB2 didn’t influence hBMSC proliferation. **e**–**g** hBMSCs were incubated with different concentrations (0–100 ng/ml) of recombinant SerpinB2 for 3 days. The mRNA expression of COL1A1 and SP7 decreased dramatically by SerpinB2. **h**–**k** The expression of COL1A1, RUNX2 and SP7 protein levels also decreased significantly with SerpinB2 (0–100 ng/ml). All data are expressed as mean ± SD. Assays were performed in triplicate. *P < 0.05; **P < 0.01; ***P < 0.001; ****P < 0.0001 compared with the control group

To ascertain the expression level of SerpinB2 associated with the osteogenesis of hBMSCs, we compared endogenous SerpinB2 expression among various differentiated stages of hBMSCs. Compared with undifferentiated hBMSCs, both of mRNA and protein expression level of SERPINB2 decreased dramatically after osteogenic differentiation on days 1 and 3 (Fig. 1a–c). To determine whether exogenous recombinant SerpinB2 influences the proliferation of hBMSCs, cell viability was detected using a Cell Counting Kit-8 (CCK8) assay after adding different concentrations of SerpinB2 (0–100 ng/ml) on days 0, 1, and 3. There is no significant difference in cell viability among different concentrations of SerpinB2 (Fig. 1d).

To explore the effects of exogenous SerpinB2 on hBMSCs differentiation, the expression levels of osteo-specific genes and proteins, including collagen type I alpha 1 (COL1A1), osterix (Osx/SP7), osteopontin (OPN), osteocalcin (OCN) and runt-related transcription factor 2 (RUNX2) were detected by western blot and quantitative real-time polymerase chain reaction (qPCR) analyses. We found that the mRNA expression levels of COL1A1 and SP7 decreased dramatically after treatment with SerpinB2 on day 3. (Fig. 1e–g). COL1A1, RUNX2, and SP7 protein levels also decreased significantly after treatment with SerpinB2 on day 3. (Fig. 1h–k).

**Fig. 2:**
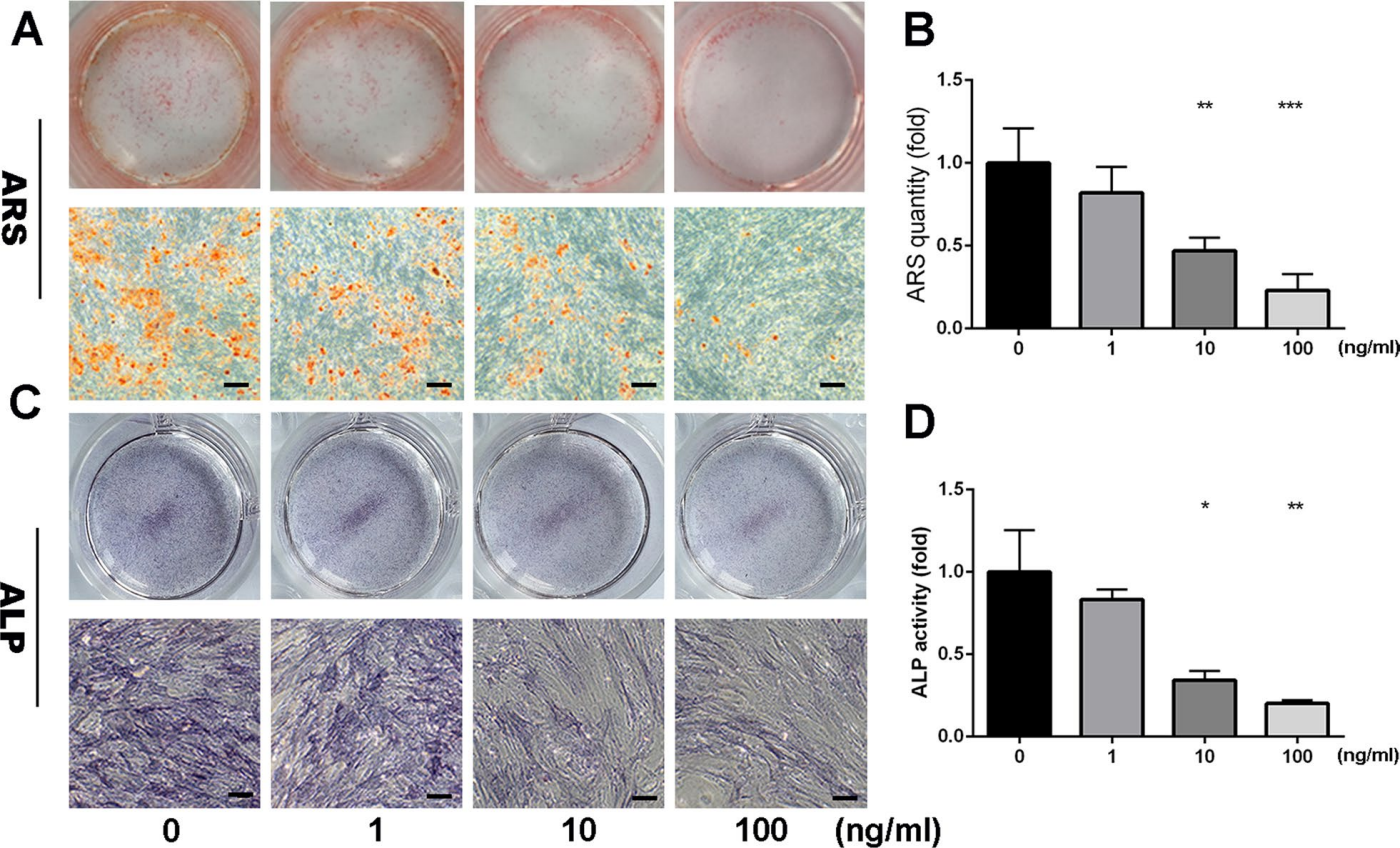
Exogenous recombinant SerpinB2 inhibited mineralization and ALP activity. **a**, **b** Recombinant SerpinB2 inhibited hBMSC mineralization. hBMSCs were incubated with different concentrations of Serpinb2 during osteogenesis for 15 days. Calcium deposits level were colored by Alizarin Red S staining. SerpinB2 inhibited mineralization significantly compared with the control group. Scale bars, 50 um. **c**, **d** Recombinant SerpinB2 inhibited the ALP activity of hBMSCs. hBMSCs were incubated with different concentrations of SerpinB2 during osteogenesis for 6 days. ALP activity was detected by ALP staining. SerpinB2 inhibited the ALP activity significantly compared with the control group. Scale bars, 50 um

We conducted Alkaline Phosphatase Live (ALP) staining, which reveals the osteogenesis level in the early phase, and ARS, which reveals the extent of calcium deposits formed during late-stage osteogenic differentiation. On day 6, SerpinB2 (0–100 ng/ml) was found to inhibit ALP activity compared with the control group, whereas treatment with SerpinB2 (0–100 ng/ml) during osteogenesis resulted in decreased the number of calcium deposits compared with the control group on day 15 (Fig. 2a–d).

### SERPINB2 knockdown increased the expression levels of osteo-specific genes and proteins, and enhanced calcium deposit formation

**Fig. 3:**
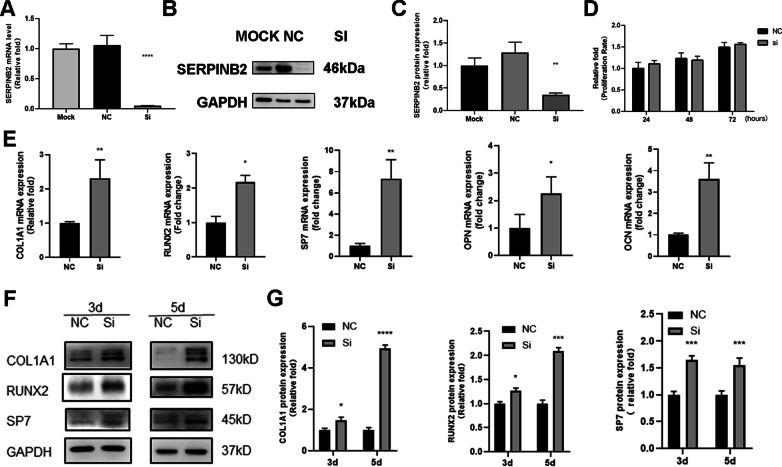
Establishment of SERPINB2 knockdown hBMSCs cell line and effects of SERPINB2 knockdown on osteogenic differentiation of hBMSCs. **a**–**c** Verification of SERPINB2 knockdown in hBMSCs. **d** SERPINB2 knockdown didn’t influence hBMSCs proliferation. **e** SERPINB2 knockdown significantly promoted the mRNA expression of COL1A1, RUNX2, SP7, OPN and OCN during osteogenesis differentiation at day 3. **f**, **g** SERPINB2 knockdown significantly promoted the protein expression level of COL1A1, RUNX2 and SP7 during osteogenesis differentiation at days 3 and 5. Data are expressed as mean ± SD. Assays were performed in triplicates. *P < 0.05; **P < 0.01; ***P < 0.001; ****P < 0.0001 compared with the control group

We then abrogated SERPINB2 gene expression in hBMSCs using small interfering RNA (siRNA). The inhibition efficiency was confirmed by qPCR and western blotting (Fig. 3a–c). To determine whether SERPINB2 knockdown influences the proliferation of hBMSCs, cell viability was determined by the CCK8 assay after SERPINB2 knockdown (Fig. 3d).

To elucidate the role of SERPINB2 knockdown in the osteogenic differentiation of hBMSCs, we determined the expression levels of osteo-specific genes and proteins, including COL1A1, OPN, OCN, RUNX2 and SP7 by western blot and qPCR analyses.

Compared with the control group, SERPINB2 knockdown significantly promoted the mRNA expression of COL1A1, OPN, OCN, RUNX2 and SP7 during osteogenesis differentiation at day 3 (Fig. 3e). Furthermore, western blotting demonstrated that the levels of COL1A1, RUNX2, and SP7 also increased remarkably following the increased mRNA expression at days 3 and 5 (Fig. 3f, g).

**Fig. 4:**
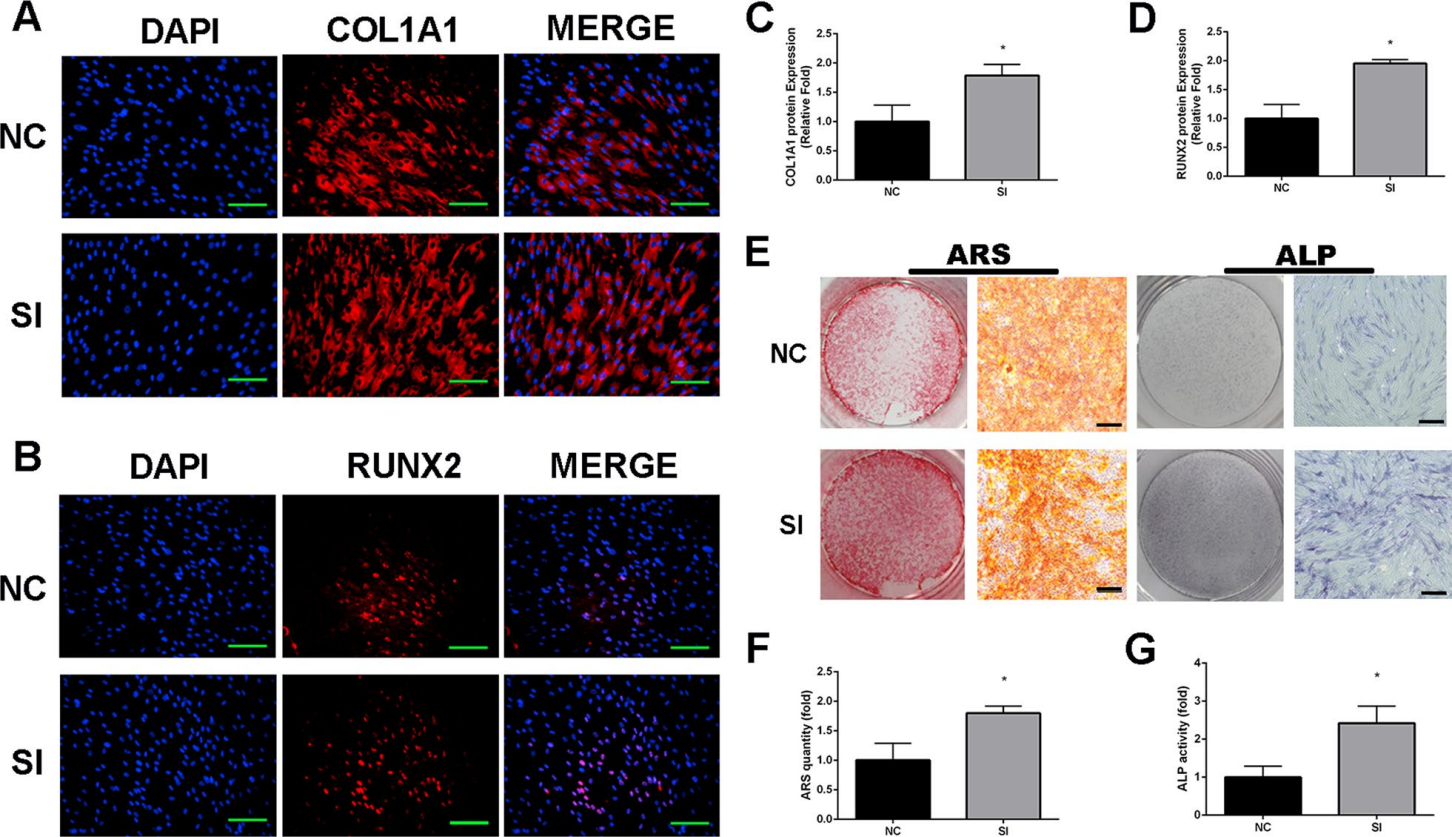
SERPINB2 gene knockdown promoted the expression of COL1A1 and RUNX2 of hBMSCs, meanwhile promoted mineralization and ALP activity of hBMSCs. **a**–**d** Immunofluorescence staining of RUNX2 and COL1A1 proteins. On day 3 of osteogenic differentiation, RUNX2 and COL1A1 (red) increased with SERPINB2 knockdown. Cell nuclei were counter-stained with DAPI (blue). Scale bars, 100 um. **e**–**g** SERPINB2 knockdown promoted hBMSCs mineralization and ALP activity. For calcium deposits level detecting, hBMSCs were cultured with osteogenic differentiation medium for 15 days. For ALP activity detecting, hBMSCs were cultured with osteogenic differentiation medium for 6 days. Data are expressed as mean ± SD. Assays were performed in triplicates. *P < 0.05; **P < 0.01; ***P < 0.001 compared with the control group

Immunofluorescence assay demonstrated that SERPINB2 knockdown promoted RUNX2 and COL1A1 protein expression in hBMSCs on day 3 (Fig. 4a–d).

ALP staining showed that SERPINB2 knockdown significantly increased ALP activity on day 6. ARS results showed that, on day 15, SERPINB2 knockdown considerably increased the numbers of calcium deposits compared to the control group (Fig. 4e–g).

### SERPINB2 knockdown promotes osteogenic differentiation of hBMSCs via the Wnt/β-catenin signaling pathway

To explore the particular signaling pathways through which SERPINB2 knockdown regulated hBMSC osteogenic differentiation, we performed western blotting to detected the expression of the common signaling pathways that involved in osteogenesis, including the phosphatidylinositol-3-kinase PI3K/AKT signaling pathway, the extracellular signal-regulated kinase/mitogen-activated protein kinase (ERK/MAPK) signaling pathway, and the Wnt/β-catenin pathway.

**Fig. 5:**
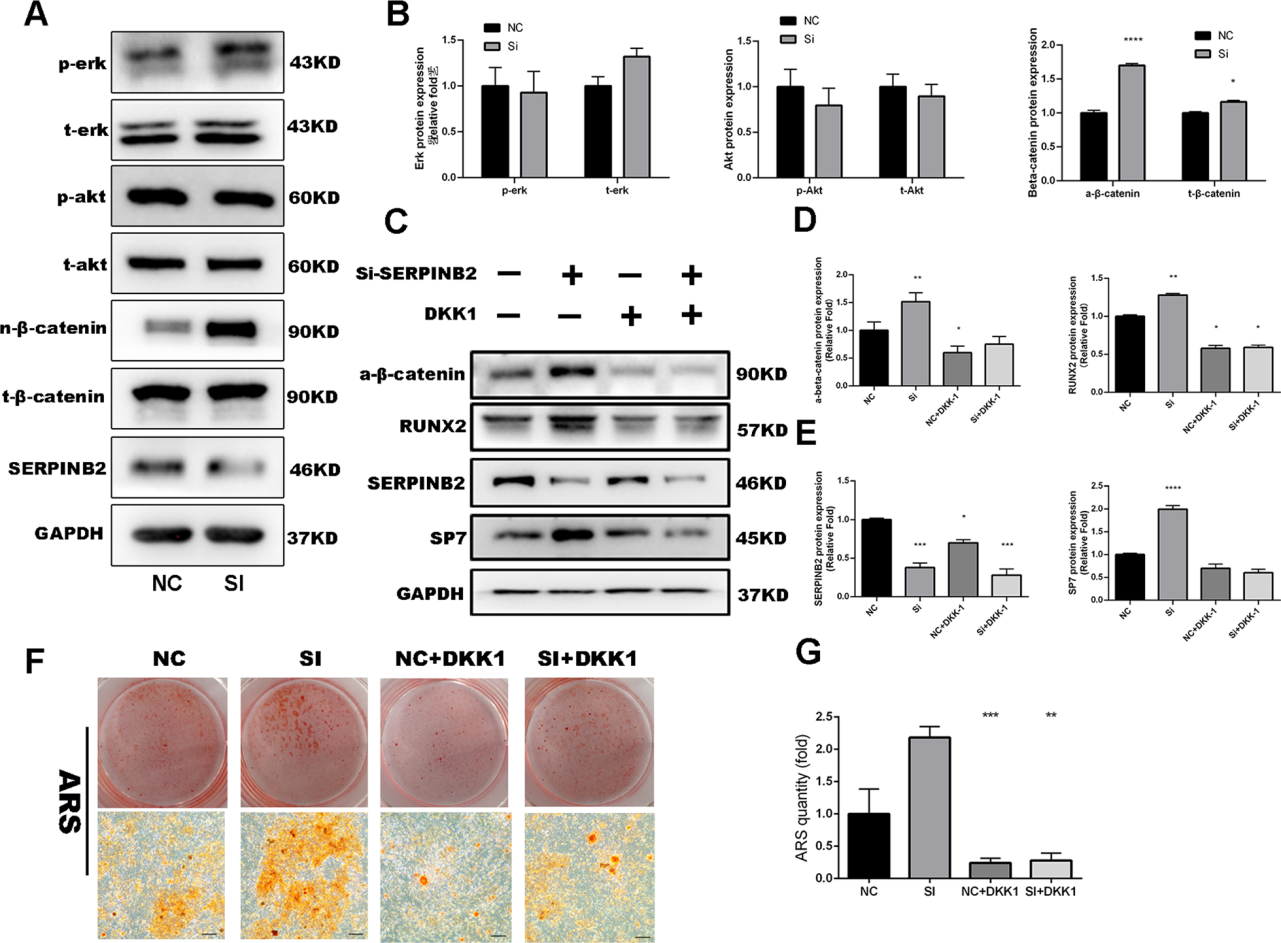
SERPINB2 knockdown of hBMSCs activated the Wnt/β-catenin signaling pathway. **a**, **b** Comparison of signaling pathway-related protein levels by Western blotting. SERPINB2 knockdown of hBMSCs upregulated the active β-catenin compared with the control group during osteogenic differentiation. Protein expression levels were normalized to that of GAPDH. **c**–**e** Increased expression of osteo-specific proteins (RUNX2 and SP7) induced by SERPINB2 knockdown was almost suppressed by DKK1. Protein expression levels were normalized to GAPDH. **f**, **g** Increased mineralization of hBMSCs by SERPINB2 knockdown was attenuated by DKK1. Scale bars, 50 um. Data are expressed as mean ± SD. Assays were performed in triplicate. *P < 0.05; **P < 0.01; ***P < 0.001; ****P < 0.0001 compared with the control group

The levels of active β-catenin increased after SERPINB2 knockdown during osteogenic differentiation, while no significant changes were observed in the ERK/MAPK signaling pathway or PI3K/AKT signaling pathway (Fig. 5a, b).

To further clear the involvement of the Wnt/β-catenin signaling pathway in the regulation of hBMSC’s osteogenic differentiation by SERPINB2 knockdown, we used Dickkopf-related protein 1 (DKK1), which is an effective inhibitor of the Wnt/β-catenin signaling pathway, with an appropriate concentration (500 ng/mL) almost complete abrogate the promotive effect on RUNX2 and COL1A1 expression induced by SERPINB2 knockdown (Fig. 5c–e).

Moreover, the ARS assay revealed that the increase in calcium deposits by SERPINB2 knockdown were suppressed by DKK1 (500 ng/mL) (Fig. 5f, g).

### SERPINB2 knockdown accelerated fracture healing in a murine tibial fracture model

To prove the roles of SERPINB2 knockdown in vivo, we developed a murine tibial fracture model. Atelocollagen (MOCK group), atelocollagen combined with negative control siRNA (NC group) and atelocollagen combined with SERPINB2 siRNA (SI group) was injected into fracture site locally respectively. The efficiency of SERPINB2 knockdown was confirmed by immunofluorescence (Additional file 1: Figure S1).

**Fig. 6:**
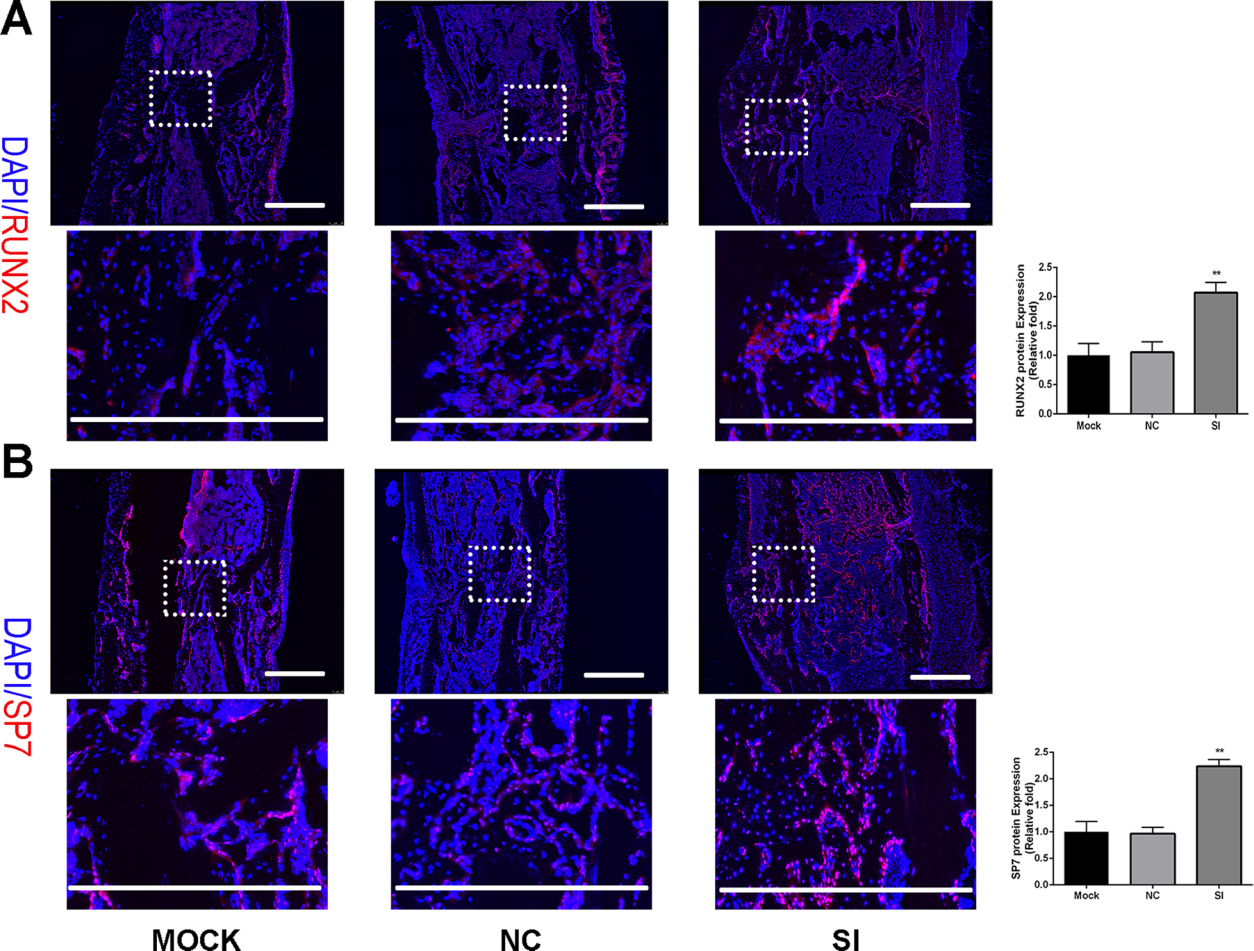
SERPINB2 knockdown in the fracture sites promoted the expression of RUNX2 and SP7. **a**, **b** Immunofluorescence analysis for bone healing. Immunofluorescence assay to asses expression of osteo-specific proteins in the fracture sites at day 14. SERPINB2 konckdown (SI) group showed increased expression of RUNX2 and SP7 compared to those in the controlled groups. Scale bars, 500 um. Data are expressed as mean ± SD. Assays were performed in triplicate. *P < 0.05; **P < 0.01; ***P < 0.001; ****P < 0.0001 compared with the control group

We used immunofluorescence assay to asses expression of osteo-specific proteins in the fracture sites at day 14. SI group showed increased expression of RUNX2 and SP7 proteins compared to those in the controlled groups (Fig. 6a, b).

**Fig. 7:**
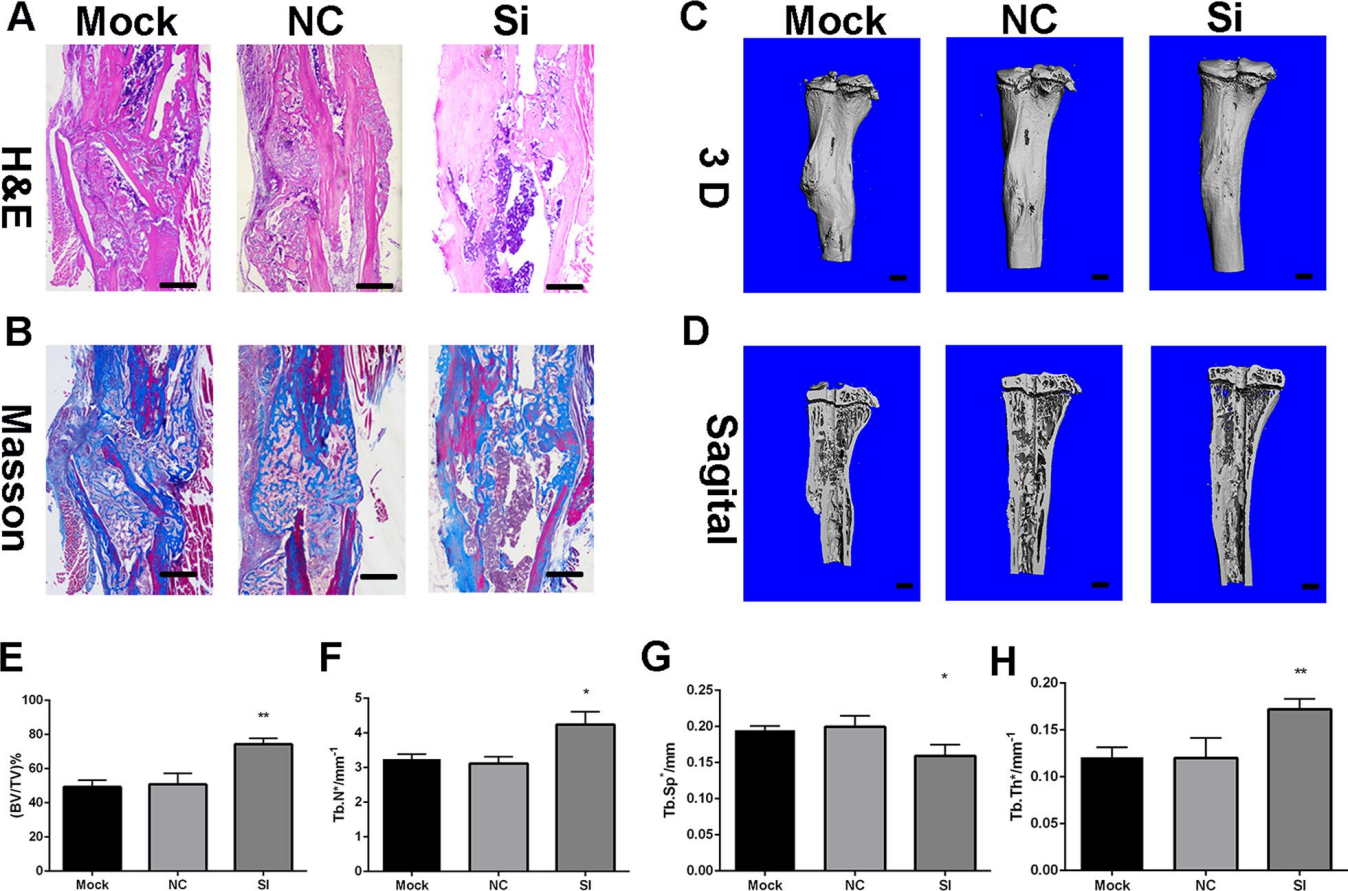
SERPINB2 knockdown accelerated bone healing in a murine tibial fracture model. **a**, **b** Histological analysis for bone healing. HE, hematoxylin and eosin staining; Masson, Masson’s trichrome staining. Scale bars, 500 um. **c**–**h** Microcomputed tomography analysis for bone healing. Scale bars, 500 um. Data are expressed as mean ± SD. Assays were performed in triplicate. *P < 0.05 compared with the NC group

We used the Masson’s trichrome stain, and hematoxylin–eosin staining to asses the remodeling of the mineralized callus of the fracture healing samples at day 14 qualitatively (Fig. 7a, b).

And to confirm this observation quantitatively, we collected fracture healing samples day 28 and those samples were analysed by microcomputed tomography (microCT). The quantitative analysis of the micro-CT data further validated the histological results that SERPINB2 knockdown promotes the fracture healing (Fig. 7c–h).

## Discussion

The plasminogen activation system has been demonstrated to play an important role in bone metabolism. Carmeliet et al. [36] generated uPA and tPA double knockout mice (tPA−/−;uPA−/−) and found that the lack of plasminogen activators increased bone formation. The elongation of bones and increased bone mass were observed, and there was a modified composition of extracellular bone matrix in tPA−/− and uPA−/− mice. In plasminogen (Plg) knockout (Plg−/−) mice, the population of pre-osteoclasts increased, and the expression of osteoprotegerin in osteoblasts decreased [34]. Overexpression of plasminogen activator inhibitor-1 increases bone mineralization in mouse femora and enhances their biomechanical properties [37]. SERPINB2 gene expression was upregulated in non-bone-forming (hBMSC^−bone^) cells compared with bone-forming cells (hBMSCs^+bone^) [35, 38]. Our results revealed that exogenous SERPINB2 inhibited the expression of osteogenic-specific genes and proteins in hBMSCs, and ALP activity and calcium deposition were also inhibited by exogenous SERPINB2. SERPINB2 gene deletion significantly promoted osteogenesis in hBMSCs.

The RUNX family of transcription factors, including Runx1, Runx2, and Runx3, characterized by a 128-amino acid long “Runt domain” [39], play an essential role in cell development. Runx2 is the master transcription factor for osteogenic differentiation of BMSCs and inhibits adipocyte differentiation [40]. Runx2 is mainly regulated by the Wnt/β-catenin signaling pathway, also known as the canonical Wnt pathway, one of the three major branches of Wnt signaling [41]. β-catenin is an essential component of the Wnt/β-catenin pathway and is the central target in the search for therapeutic agents [42–44].

Wnt ligands are a large family of cysteine-rich glycoproteins that control various biological processes in various cell types [45, 46]. The Wnt receptors include Frizzled family members and low-density lipoprotein receptor-related proteins 5 and 6 (LRP5/6) [47, 48]. In the absence of Wnt ligands, cytoplasmic β-catenin is phosphorylated by the Axin complex, which is composed of Axin, adenomatous polyposis coli, glycogen synthase kinase 3β, and casein kinase 1, and then degraded by the proteasome, maintaining the repression of Wnt target genes inhibited by DNA-bound T cell factor/lymphoid enhancer factor (TCF/LEF). Once Wnt binds to the Frizzled receptor and its co-receptor LRP5/6, the cytoplasmic tail of the LRPs binds to the phosphorylated site of Axin, and cytoplasmic β-catenin is released from the Axin complex and translocated into the nucleus, and initiates its target genes by forming complexes with TCF/LEF.

In our study, the active-β-catenin expression levels decreased in response to exogenous SerpinB2 during osteogenic differentiation and increased dramatically when the SERPINB2 gene was deleted. Moreover, the effects of SERPINB2 silencing on osteogenesis were almost completely abrogated by DKK1, a catenin-dependent Wnt signaling pathway inhibitor. These results indicate that SERPINB2 silencing promotes osteogenic differentiation of hBMSCs through the Wnt/β-catenin signaling pathway.

However, some problems require further investigation. First, in our study, SERPINB2 siRNA combined with atelocollagen was injected locally into the fracture site, and SERPINB2 silencing successfully accelerated bone healing in a murine tibial fracture model. With the development of biomaterials, an increasing number of drugs are being released into controlled and target tissues. Thus, further studies should focus on the more efficient delivery of SERPINB2 siRNA with the help of biomaterials. Second, SERPINB2 was found to be extracellular. The skeleton not only performs its structural role but has also been found to be an endocrine organ linked to other organs. Thus, it is worth investigating whether SERPINB2 secreted by other organs influences the homeostasis of the skeletal system.

## Conclusion

Taken together, these findings indicate that SERPINB2 knockdown promotes osteogenic differentiation of hBMSCs through upregulate the active β-catenin, and effectively promotes fracture healing in a murine fracture model.

## Reagents and methods

### Reagents

hBMSCs were purchased from Cyagen Biosciences (Guangzhou, China). hBMSC growth medium were purchased from Cyagen Biosciences. Cells cultured in a atmosphere of 5% CO_2_ at 37 °C. Osteogenic induction medium was prepared according to previous methods [49].

SerpinB2 recombinant protein was purchased from Amyjet Scientific (Abnova, wuhan, china). Antibodies used for western blotting including GAPDH (1:1000, Beyotime, shanghai, china), RUNX2 (1:1000, Beyotime), COL1A1 (1:1000, Beyotime), SERPINB2 (1:1000, Abcam), SP7/Osterix (1:1000, Abcam, shanghai, china), non-phosphorylated (active) β-catenin (1:1000; Cell Signaling Technology), or total β-catenin (1: 1000; Beyotime), phosphorylated and total ERK (1:1000; Cell Signaling Technology), phosphorylated and total AKT (1:1000; Cell Signaling Technology). Second antibodies Alexa Fluor 555 was purchased from Beyotime. Lipo6000 Transfection Reagents were purchased from Beyotime (Shanghai, china).

The siRNAs were purchased from Genepharma (Hangzhou, china).

hSerpinb2 sense: GCAAAGAAUCAAGUUGCAATT

hSerpinb2 antisense: UUGCAACUUGAUUCUUUGCTT

hNC sense: UUCUCCGAACGUGUCACGUTT

hNC antisense: TTAAGAGGCUUGCACAGUGCA

mSerpinb2 sense: GGAGAGAAGUCUGCAAGAUTT

mSerpinb2 antisense: AUCUUGCAGACUUCUCUCCTT

mNC sense: GGCUCUGTTCUUGUCACGUAA

mNC antisense: UUTTGAGGCAAGCACAGUGCA

### Cell proliferation assay

Cells were cultured in 96-well plate at a density of 5000 cells per well, CCK-8 (Beyotime) of 10% was added into wells and incubated with cells for 2 h at 37 °C. Then the cell proliferation was measured by a microplate reader at the absorbance of 450 nm (BioTek, ELX808).

### RT-qPCR

Total RNA from cells were extracted with RNA isolation reagent (Takara) and RNA was quantified by spectrophotometer at 260 nm wave length (NanoDrop 2000; ThermoFisher). Total RNA was then reverse-transcribed with the Double-Strand cDNA Synthesis reagent (Takara) and cDNA (2 ul) was used to quantification using SYBR Green PCR Master Mix reagent (Takara), and finally detected by a StepOnePlus System (Applied Biosystems). The reaction conditions were as previous described: two microliter cDNA was used for qPCR. mRNAs of target genes or the housekeeping gene (GAPDH OR 18S) was quantified in separate tubes. All primers of genes were synthesized by GENEray (shanghai, China). The cycle conditions were as follows: 95 °C for 30 s, then 95 °C for 5 s for 40 cycles and 60 °C for 30 s. The relative target gene expression levels were calculated using the 2^−ΔΔCt^ method.

### Western blot analysis

Proteins was extracted from cells by lysing in RIPA buffer supplemented with protease inhibitors and phosphatase inhibitors (Beyotime). After protein quantification, cell lysates were suspended in 5 X loading buffer and then equal amounts of proteins were subjected to migration on 10–12% polyacrylamide gels, then transferred to a polyvinylidene fluoride membrane (Millipore, Shanghai, China). The membranes were blocked by non-fat milk (5%) for 60 min and then incubated with primary antibodies for 12–16 h at 4 °C. After, membranes were washed with Tris-buffered saline-Tween (TBST) 3 times (10 min each) and incubated with HRP-conjugated secondary antibodies (Beyotime) for 1 h at RT. Finally, proteins were revealed by chemiluminescence reagents (Millipore) and the signal intensity was detected using Bio-Rad XRS system (Bio-Rad). Results were normalized to total protein concentration.

### ALP staining

Cells were passaged to 12-well plates and cultured with osteogenic induction medium when cells reached at 70% confluence. Osteogenic induction medium cultured for 5 days, cells were washed by PBS 3 times, and were fixed in 4% paraformaldehyde for 20–30 min at RT. Washed by PBS 3 times, cells were stained by the ALP Color Development Kit (Beyotime). To measure ALP quantified, ALP activity was determined by ALP Activity Assay (Beyotime). Stain was lysed with lysis buffer consisted of 20 mM Tris–HCl (pH 7.5), 1% Triton X-100 and 150 mM NaCl and the conversion color of p-nitrophenyl phosphate was measured at 405/650 nm by a microplate reader (ELX808; BioTek).

### Alizarin red staining

Cells were passaged to 12-well plates and cultured with osteogenic induction medium when cells reached at 70% confluence. Osteogenic induction medium cultured for 15 days, cells were washed by PBS 3 times and were fixed in 4% paraformaldehyde for 20–30 min at RT. Washed by PBS for 3 times, calcium deposition was stained by Alizarin Red staining (Cyagen) for 5–10 min at RT, and then was rinsed by PBS 3 times. The ARS quantification was performed as previous described. In a brief, the stain incubated with 10% cetylpyridinium chloride for 1 h at RT, solutions were collected, then 200 μl solutions were plated on a 96-well plate and were read at 560 nm by a microplate reader (ELX808; BioTek). Results were normalized to controlled group.

### siRNA transfection

Cells were seeded in plates and transfected with siRNA when cells reached 30–50% confluence. Cells were transfected with Lipo6000 Transfection Reagent (MOCK group), Lipo6000 Transfection Reagent combined with NC siRNA (NC group), Lipo6000 Transfection Reagent combined with target gene siRNA (SI group). Transfection process was according to the manufacturer’s instructions. Transfection efficiency was verified by qPCR.

### Animals

All C57/B16 mice (male, 12 weeks) were purchased from SLAC Laboratory Animal Co (Shanghai, China). All of the animal experiments were approved by the Institutional Animal Care and Use Committee of the 2nd Affiliated Hospital, Zhejiang University (No: 2020-1035). All mice were randomly divided into control and experimental groups.

Sample sizes used at minimum 5 animals per experimental procedure.

#### Mice-fracture model

Mice-fracture model was modified on the basis of previous method [50]. Briefly, mice anesthetized by intraperitoneally injection of 0.3% pentobarbital sodium (30 mg/kg body). Exposed the right lower limb, made an incision lateral between the middle of tibia tuberosity and crest. A 0.38-mm diameter intramedullary fixation pin was then inserted into the tibia’s medullary canal at the level of the tibia tuberosity for fixation. Separated the soft tissue carefully and stripped the periosteum above the crest of tibia. Then an osteotomy was created above the crest of tibia. The same leg was used in each group.

For in vivo study of SERPINB2 silence effects, animals were divided into three groups. Atelocollagen, atelocollagen combined with negative control siRNA (NC-SI) and atelocollagen combined with mice SERPINB2 siRNA (SE-SI) was injected into fracture site locally respectively. 2 and 4 weeks after surgery, limbs were harvested by lethal intraperitoneal injection of 0.1 ml sodium pentobarbitone (200 mg/ml) for next experiments.

### Histology

Following harvest, 2 weeks of samples were fixed by 10% paraformaldehyde for 36 h at 4 °C and then were decalcified by 0.5 M ethylene diaminetetra acetic acid (EDTA, Beyotime) for 3 days at 4 °C. Specimens were then embedded in paraffin and sectioned at a 5um thickness. Serial sections were deparaffinized and then stained with hematoxylin–eosin staining, Masson’s Trichrome according to the standard procedures.

### Histology immunofluorescence analysis

For mouse sample, following harvest, 2 weeks of samples were fixed by 10% paraformaldehyde for 4 h at 4 °C and then were decalcified by 0.5 M EDTA (Beyotime) for 3 days at 4 °C. Freshly dissected bone tissues and immunofluorescent stainings were according to previous methods described by Adams et al. [35]. The following primary antibodies were used: Sp7/Osterix (abcam, diluted 1:100), RUNX2 (abcam, diluted 1:100). Target protein was observed under a fluorescence microscope (Leica) and quantified by Image J software.

### Radiographic analysis

Following harvest, 4 weeks of samples were sent to make a microcomputed tomography (μCT) evaluation. Each tibia was scanned using μCT-100 imaging system (Scanco Medical, Switzerland), operation parameters were according to the previous report [51].

### Statistical analysis

All experiments were performed at least in triplicate, data are presented as means ± SD, statistical significance between two groups was determined by Student’s t test, one-way ANOVA or Bonferroni’s post-hoc test according to the distribution of the tested population. Statistical analysis was performed with SPSS 19.0 software (IBM, USA). A value of *P* ≤ 0.05 was considered significant.

## Supplementary Information

**Additional file 1: Fig. S1.** The efficiency of SERPINB2 knockdown. The efficiency of SERPINB2 knockdown in fracture sites was confirmed by Immunofluorescence assay. Scale bars, 500 um. Data are expressed as mean±SD. Assays were performed in triplicate. *, P < 0.05, **, P<0.01 compared with the control group.

## Data Availability

The datasets used and/or analyzed during the current study are available from the corresponding author on reasonable request.

## Abbreviations

SERPINB2: Serpin family B member 2
PAI-2: Plasminogen activator inhibitor type 2
GAPDH: Glyceraldehyde-3-phosphate dehydrogenase
RUNX2: Runt-related transcription factor 2
COL1A1: Alpha-1 type I collagen
OCN: Osteocalcin
Osx/SP7: Osterix
OPN: Osteopontin
ALP: Alkaline phosphatase
ARS: Alizarin red staining
uPA: Urokinase plasminogen activator
tPA: Tissue plasminogen activator
LRP5/6: Lipoprotein receptor-related proteins 5 and 6
BMSCs: Human bone marrow mesenchymal stem cells
IF: Immunofluorescence
hBMSC^−bone^: Non-bone-forming human bone marrow mesenchymal stem cells
hBMSCs^+^^bone^: Bone-forming human bone marrow mesenchymal stem cells
