# Supplementary figures and images for: Knockdown of SERPINB2 enhances the osteogenic differentiation of human bone marrow mesenchymal stem cells via activation of the Wnt/β-catenin signalling pathway
Source: Stem Cell Res Ther. 2021 Oct 7;12:525. doi: 10.1186/s13287-021-02581-6 (PMC8499504; doi:10.1186/s13287-021-02581-6)

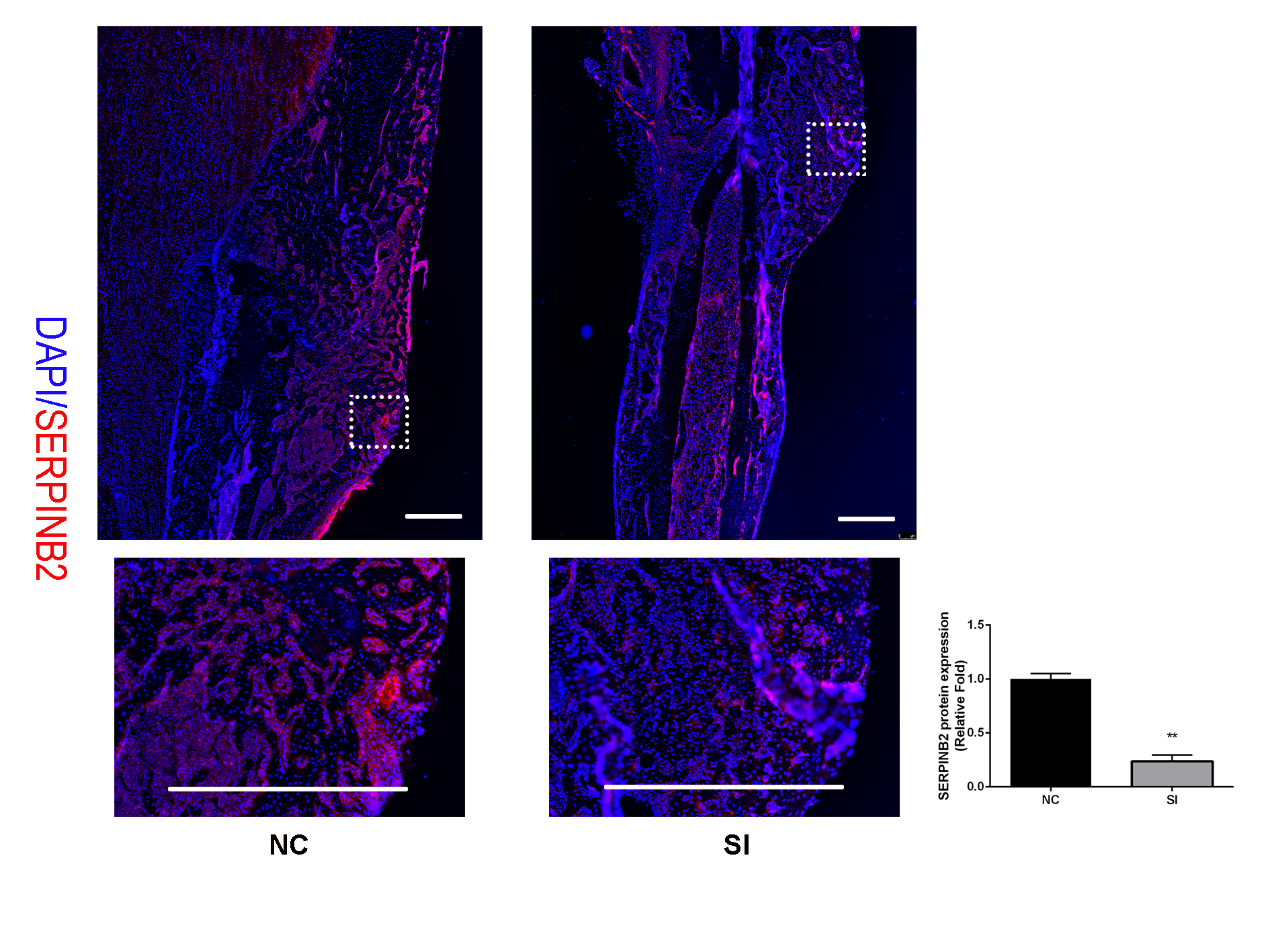

Supplement: Supplementary file 1 — Additional file 1: Fig. S1. The efficiency of SERPINB2 knockdown. The efficiency of SERPINB2 knockdown in fracture sites was confirmed by Immunofluorescence assay. Scale bars, 500 um. Data are expressed as mean±SD. Assays were performed in triplicate. *, P < 0.05, **, P<0.01 compared with the control group. [file 13287_2021_2581_MOESM1_ESM.png]
